# Bioresmethrin: (5-benzyl­furan-3-yl)methyl 2,2-dimethyl-3-(2-methyl­prop-1-en-1-yl)cyclo­propane-1-carboxyl­ate

**DOI:** 10.1107/S1600536812040767

**Published:** 2012-10-03

**Authors:** Tae Ho Kim, Ki-Min Park, Youngeun Jeon, Jineun Kim

**Affiliations:** a Department of Chemistry and Research Institute of Natural Sciences, Gyeongsang National University, Jinju 660-701, Republic of Korea

## Abstract

In the title compound, C_22_H_26_O_3_, the dihedral angle between the cyclo­propane ring and the plane of the vinyl group is 88.2 (2)°. The dihedral angle between the phenyl and furan rings is 86.09 (8)°. In the crystal, weak inter­molecular C—H⋯π contacts together with very weak C—H⋯O hydrogen bonds stack the mol­ecules along the *a* axis.

## Related literature

For information on the insecticidal activity of the title compound, see: Hill *et al.* (1993 ▶). For a related structure, see: Yang *et al.* (2011 ▶).
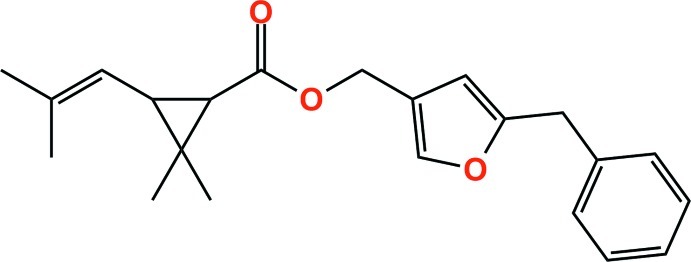


## Experimental

### 

#### Crystal data

C_22_H_26_O_3_
*M* *_r_* = 338.43
Monoclinic, 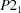

*a* = 7.8438 (14) Å
*b* = 11.555 (2) Å
*c* = 10.9649 (18) Å
β = 108.375 (3)°
*V* = 943.2 (3) Å^3^
*Z* = 2
Mo *K*α radiation
μ = 0.08 mm^−1^
*T* = 173 K
0.33 × 0.28 × 0.10 mm

#### Data collection

Bruker APEXII CCD diffractometer
Absorption correction: multi-scan (*SADABS*; Sheldrick, 1996 ▶) *T*_min_ = 0.975, *T*_max_ = 0.992
9877 measured reflections
2458 independent reflections
2156 reflections with *I* > 2σ(*I*)
*R*_int_ = 0.058

#### Refinement

*R*[*F* ^2^ > 2σ(*F* ^2^)] = 0.043
*wR*(*F* ^2^) = 0.096
*S* = 1.04
2458 reflections
230 parameters
1 restraint
H-atom parameters constrained
Δρ_max_ = 0.20 e Å^−3^
Δρ_min_ = −0.17 e Å^−3^

### 

Data collection: *APEX2* (Bruker, 2006 ▶); cell refinement: *SAINT* (Bruker, 2006 ▶); data reduction: *SAINT*; program(s) used to solve structure: *SHELXTL* (Sheldrick, 2008 ▶); program(s) used to refine structure: *SHELXTL*; molecular graphics: *SHELXTL* and *DIAMOND* (Brandenburg, 1998 ▶); software used to prepare material for publication: *SHELXTL*.

## Supplementary Material

Crystal structure: contains datablock(s) global, I. DOI: 10.1107/S1600536812040767/sj5266sup1.cif

Structure factors: contains datablock(s) I. DOI: 10.1107/S1600536812040767/sj5266Isup2.hkl

Supplementary material file. DOI: 10.1107/S1600536812040767/sj5266Isup3.cml

Additional supplementary materials:  crystallographic information; 3D view; checkCIF report

## Figures and Tables

**Table 1:** Hydrogen-bond geometry (Å, °) *Cg* is the centroid of the C1–C6 phenyl ring.

*D*—H⋯*A*	*D*—H	H⋯*A*	*D*⋯*A*	*D*—H⋯*A*
C7—H7*A*⋯O3^i^	0.99	2.71	3.516 (3)	139
C11—H11⋯*Cg* ^ii^	0.95	2.63	3.559 (3)	167

Symmetry codes: (i) 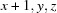; (ii) 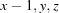.

**Fig. 1.**
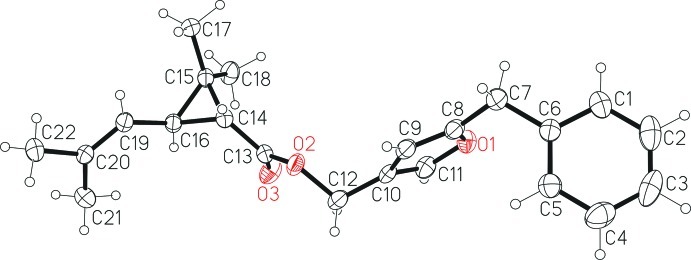
The molecular structure of the title compound. Displacement ellipsoids are drawn at the 50% probability level. H atoms are shown as small spheres of arbitrary radius.

**Fig. 2.**
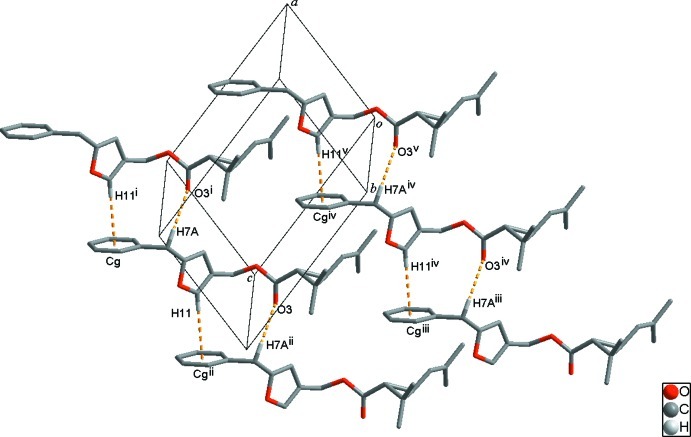
Crystal packing of the title compound with weak intermolecular C—H···π and C–H···O interactions shown as dashed lines. H atoms not involved in intermolecular interactions have been omitted for clarity. (Symmetry codes: (i) x + 1, y, z; (ii) x - 1, y, z; (iii) x - 2, y, z - 1; (iv) x - 1, y, z - 1; (v) x, y, z - 1.

## supplementary crystallographic information

### Comment

Bioresmethrin (systematic name: 5-benzyl-3-furylmethyl- 2,2-dimethyl-3-(2-methylprop-1-enyl)cyclopropanecarboxylate), is a synthetic pyrethroid with high insecticidal activity aganist a wide range of insect pests (Hill *et al.*, 1993). However its crystal structure has not yet been reported.

In the title compound (Scheme 1, Fig. 1), the dihedral angle between the cyclopropane ring plane and the vinyl group plane is 88.2 (2)°. The dihedral angle between the benzene and furan ring planes in the benzylfuranyl group is 86.09 (8)°. All bond lengths and bond angles are normal and comparable to those observed in a similar crystal structure (Yang *et al.*, 2011).

In the crystal structure (Fig. 2) weak intermolecular C—H···π interactions [C11···*Cg*^ii^ 3.559 (4) Å. *Cg* is the centroid of the C1—C6 ring. (Symmetry codes: (ii) *x* - 1, *y*, *z*) are found together with very weak C7–H7A···O3 hydrogen bonds, Table 1, stack the molecules along *a*. These intermolecular interactions may contribute to the stabilization of the packing.

### Experimental

The title compound was purchased from the Dr. Ehrenstorfer GmbH Company. Slow evaporation of a solution in CH_2_Cl_2_ gave single crystals suitable for X-ray analysis.

### Refinement

All H-atoms were positioned geometrically and refined using a riding model with d(C—H) = 1.00 Å, *U*_iso_ = 1.2*U*_eq_(C) for methine C—H, d(C—H) = 0.95 Å, *U*_iso_ = 1.2*U*_eq_(C) for C*sp*^2^—H and d(C—H) = 0.98 Å, *U*_iso_ = 1.5*U*_eq_(C) for CH_3_ groups. In the absence of significant anomalous scattering effects, Friedel pairs were merged. Because of this the absolute configuration of the title compound could not be reliably determined from the crystallographic data but has been suggested to be 5-benzyl-3-furylmethyl (1*R*,3*R*)- 2,2-dimethyl-3-(2-methylprop-1-enyl)cyclopropanecarboxylate from information provided by the manufacturers (the Dr. Ehrenstorfer GmbH Company). However, this cannot be confirmed by the present crystallographic determination.

### Crystal data

**Table:**

C_22_H_26_O_3_	*F*(000) = 364
*M**_r_* = 338.43	*D*_x_ = 1.192 Mg m^−^^3^
Monoclinic, *P*2_1_	Mo *K*α radiation, λ = 0.71073 Å
Hall symbol: P 2yb	Cell parameters from 2685 reflections
*a* = 7.8438 (14) Å	θ = 2.6–26.3°
*b* = 11.555 (2) Å	µ = 0.08 mm^−^^1^
*c* = 10.9649 (18) Å	*T* = 173 K
β = 108.375 (3)°	Plate, colourless
*V* = 943.2 (3) Å^3^	0.33 × 0.28 × 0.10 mm
*Z* = 2	

### Data collection

**Table:**

Bruker APEXII CCD diffractometer	2458 independent reflections
Radiation source: fine-focus sealed tube	2156 reflections with *I* > 2σ(*I*)
Graphite monochromator	*R*_int_ = 0.058
φ and ω scans	θ_max_ = 28.3°, θ_min_ = 2.0°
Absorption correction: multi-scan (*SADABS*; Sheldrick, 1996)	*h* = −10→10
*T*_min_ = 0.975, *T*_max_ = 0.992	*k* = −15→15
9877 measured reflections	*l* = −13→14

### Refinement

**Table:**

Refinement on *F*^2^	Primary atom site location: structure-invariant direct methods
Least-squares matrix: full	Secondary atom site location: difference Fourier map
*R*[*F*^2^ > 2σ(*F*^2^)] = 0.043	Hydrogen site location: inferred from neighbouring sites
*wR*(*F*^2^) = 0.096	H-atom parameters constrained
*S* = 1.04	*w* = 1/[σ^2^(*F*_o_^2^) + (0.0305*P*)^2^ + 0.1943*P*] where *P* = (*F*_o_^2^ + 2*F*_c_^2^)/3
2458 reflections	(Δ/σ)_max_ = 0.001
230 parameters	Δρ_max_ = 0.20 e Å^−^^3^
1 restraint	Δρ_min_ = −0.17 e Å^−^^3^

### Special details

**Table:**

Geometry. All e.s.d.'s (except the e.s.d. in the dihedral angle between two l.s. planes) are estimated using the full covariance matrix. The cell e.s.d.'s are taken into account individually in the estimation of e.s.d.'s in distances, angles and torsion angles; correlations between e.s.d.'s in cell parameters are only used when they are defined by crystal symmetry. An approximate (isotropic) treatment of cell e.s.d.'s is used for estimating e.s.d.'s involving l.s. planes.
Refinement. Refinement of *F*^2^ against ALL reflections. The weighted *R*-factor *wR* and goodness of fit *S* are based on *F*^2^, conventional *R*-factors *R* are based on *F*, with *F* set to zero for negative *F*^2^. The threshold expression of *F*^2^ > σ(*F*^2^) is used only for calculating *R*-factors(gt) *etc*. and is not relevant to the choice of reflections for refinement. *R*-factors based on *F*^2^ are statistically about twice as large as those based on *F*, and *R*- factors based on ALL data will be even larger.

### Fractional atomic coordinates and isotropic or equivalent isotropic displacement parameters (Å^2^)

**Table:**

	*x*	*y*	*z*	*U*_iso_*/*U*_eq_	
O1	0.6817 (2)	−0.05992 (14)	1.05641 (15)	0.0323 (4)	
O2	0.2777 (2)	0.11355 (14)	0.72431 (15)	0.0326 (4)	
O3	0.0111 (2)	0.09696 (17)	0.75770 (16)	0.0393 (4)	
C1	1.1967 (3)	−0.1520 (2)	1.1670 (3)	0.0378 (6)	
H1	1.1775	−0.2312	1.1430	0.045*	
C2	1.3371 (4)	−0.1216 (3)	1.2738 (3)	0.0491 (7)	
H2	1.4143	−0.1797	1.3230	0.059*	
C3	1.3657 (4)	−0.0084 (3)	1.3093 (3)	0.0531 (8)	
H3	1.4630	0.0121	1.3830	0.064*	
C4	1.2540 (4)	0.0764 (3)	1.2387 (3)	0.0499 (7)	
H4	1.2731	0.1552	1.2642	0.060*	
C5	1.1138 (3)	0.0463 (2)	1.1306 (3)	0.0363 (6)	
H5	1.0381	0.1048	1.0810	0.044*	
C6	1.0830 (3)	−0.0685 (2)	1.0940 (2)	0.0286 (5)	
C7	0.9302 (3)	−0.1018 (2)	0.9759 (2)	0.0354 (5)	
H7A	0.9666	−0.0862	0.8989	0.042*	
H7B	0.9087	−0.1860	0.9787	0.042*	
C8	0.7591 (3)	−0.03974 (19)	0.9623 (2)	0.0281 (5)	
C9	0.6617 (3)	0.03791 (19)	0.8776 (2)	0.0280 (5)	
H9	0.6877	0.0674	0.8045	0.034*	
C10	0.5105 (3)	0.06772 (19)	0.9187 (2)	0.0274 (5)	
C11	0.5305 (3)	0.0060 (2)	1.0258 (2)	0.0320 (5)	
H11	0.4492	0.0080	1.0742	0.038*	
C12	0.3638 (3)	0.1493 (2)	0.8562 (2)	0.0365 (6)	
H12A	0.2748	0.1501	0.9032	0.044*	
H12B	0.4128	0.2284	0.8581	0.044*	
C13	0.1008 (3)	0.08949 (18)	0.6877 (2)	0.0259 (4)	
C14	0.0392 (3)	0.05617 (17)	0.5514 (2)	0.0240 (4)	
H14	0.1309	0.0644	0.5059	0.029*	
C15	−0.1023 (3)	−0.03703 (18)	0.4976 (2)	0.0255 (4)	
C16	−0.1542 (3)	0.08798 (18)	0.4709 (2)	0.0244 (4)	
H16	−0.2295	0.1201	0.5214	0.029*	
C17	−0.0750 (3)	−0.1108 (2)	0.3920 (2)	0.0316 (5)	
H17A	−0.0064	−0.1802	0.4294	0.047*	
H17B	−0.0087	−0.0666	0.3454	0.047*	
H17C	−0.1920	−0.1337	0.3325	0.047*	
C18	−0.1790 (3)	−0.1031 (2)	0.5866 (2)	0.0347 (5)	
H18A	−0.2054	−0.0493	0.6474	0.052*	
H18B	−0.0916	−0.1608	0.6340	0.052*	
H18C	−0.2899	−0.1420	0.5363	0.052*	
C19	−0.1846 (3)	0.14189 (19)	0.3437 (2)	0.0267 (4)	
H19	−0.1285	0.1057	0.2885	0.032*	
C20	−0.2821 (3)	0.23548 (19)	0.2982 (2)	0.0276 (5)	
C21	−0.3813 (4)	0.3044 (2)	0.3695 (3)	0.0390 (6)	
H21A	−0.3744	0.2650	0.4501	0.058*	
H21B	−0.5075	0.3120	0.3167	0.058*	
H21C	−0.3273	0.3815	0.3885	0.058*	
C22	−0.2964 (4)	0.2819 (2)	0.1675 (2)	0.0387 (6)	
H22A	−0.2335	0.2299	0.1254	0.058*	
H22B	−0.2417	0.3590	0.1762	0.058*	
H22C	−0.4232	0.2871	0.1156	0.058*	

### Atomic displacement parameters (Å^2^)

**Table:**

	*U*^11^	*U*^22^	*U*^33^	*U*^12^	*U*^13^	*U*^23^
O1	0.0341 (8)	0.0369 (9)	0.0243 (9)	0.0015 (7)	0.0069 (7)	0.0039 (7)
O2	0.0254 (8)	0.0411 (9)	0.0249 (9)	0.0036 (7)	−0.0015 (7)	−0.0062 (7)
O3	0.0382 (9)	0.0543 (11)	0.0258 (9)	−0.0059 (8)	0.0110 (8)	−0.0055 (8)
C1	0.0355 (13)	0.0412 (13)	0.0361 (15)	0.0070 (11)	0.0105 (12)	0.0076 (11)
C2	0.0380 (15)	0.073 (2)	0.0338 (16)	0.0106 (14)	0.0083 (12)	0.0147 (15)
C3	0.0280 (13)	0.094 (3)	0.0340 (17)	−0.0070 (15)	0.0048 (12)	−0.0053 (16)
C4	0.0377 (14)	0.0526 (17)	0.0597 (19)	−0.0127 (13)	0.0159 (14)	−0.0167 (15)
C5	0.0314 (12)	0.0364 (12)	0.0398 (15)	0.0012 (10)	0.0090 (11)	−0.0013 (11)
C6	0.0264 (10)	0.0353 (11)	0.0256 (12)	0.0022 (9)	0.0105 (9)	−0.0010 (10)
C7	0.0353 (12)	0.0376 (12)	0.0296 (13)	0.0068 (10)	0.0051 (10)	−0.0053 (10)
C8	0.0282 (11)	0.0307 (11)	0.0235 (12)	−0.0021 (9)	0.0054 (9)	−0.0049 (9)
C9	0.0298 (11)	0.0297 (11)	0.0223 (12)	−0.0029 (9)	0.0051 (9)	−0.0036 (9)
C10	0.0248 (10)	0.0294 (11)	0.0222 (12)	−0.0033 (8)	−0.0008 (9)	−0.0067 (9)
C11	0.0267 (11)	0.0408 (13)	0.0279 (13)	−0.0030 (9)	0.0079 (10)	−0.0036 (10)
C12	0.0348 (13)	0.0372 (13)	0.0297 (14)	0.0040 (10)	−0.0011 (11)	−0.0121 (11)
C13	0.0269 (10)	0.0230 (9)	0.0245 (11)	0.0024 (8)	0.0034 (9)	0.0028 (9)
C14	0.0231 (10)	0.0260 (10)	0.0226 (11)	0.0016 (8)	0.0066 (9)	0.0022 (8)
C15	0.0255 (10)	0.0257 (10)	0.0223 (11)	0.0012 (8)	0.0032 (9)	0.0024 (9)
C16	0.0239 (10)	0.0267 (10)	0.0216 (11)	0.0026 (8)	0.0056 (8)	0.0007 (9)
C17	0.0377 (13)	0.0253 (10)	0.0271 (13)	0.0009 (9)	0.0034 (10)	−0.0034 (9)
C18	0.0347 (12)	0.0370 (12)	0.0297 (13)	−0.0079 (10)	0.0064 (10)	0.0055 (10)
C19	0.0275 (10)	0.0291 (10)	0.0216 (11)	0.0023 (8)	0.0051 (9)	0.0002 (9)
C20	0.0271 (11)	0.0265 (10)	0.0240 (12)	−0.0010 (8)	0.0008 (9)	−0.0009 (9)
C21	0.0406 (13)	0.0316 (12)	0.0401 (15)	0.0083 (10)	0.0060 (12)	0.0001 (11)
C22	0.0491 (15)	0.0314 (12)	0.0288 (14)	0.0030 (11)	0.0026 (11)	0.0070 (10)

### Geometric parameters (Å, º)

**Table:**

O1—C11	1.360 (3)	C12—H12B	0.9900
O1—C8	1.372 (3)	C13—C14	1.470 (3)
O2—C13	1.346 (3)	C14—C15	1.525 (3)
O2—C12	1.450 (3)	C14—C16	1.540 (3)
O3—C13	1.197 (3)	C14—H14	1.0000
C1—C2	1.377 (4)	C15—C16	1.504 (3)
C1—C6	1.385 (3)	C15—C18	1.505 (3)
C1—H1	0.9500	C15—C17	1.508 (3)
C2—C3	1.363 (5)	C16—C19	1.476 (3)
C2—H2	0.9500	C16—H16	1.0000
C3—C4	1.379 (5)	C17—H17A	0.9800
C3—H3	0.9500	C17—H17B	0.9800
C4—C5	1.383 (4)	C17—H17C	0.9800
C4—H4	0.9500	C18—H18A	0.9800
C5—C6	1.384 (3)	C18—H18B	0.9800
C5—H5	0.9500	C18—H18C	0.9800
C6—C7	1.511 (3)	C19—C20	1.327 (3)
C7—C8	1.486 (3)	C19—H19	0.9500
C7—H7A	0.9900	C20—C21	1.495 (3)
C7—H7B	0.9900	C20—C22	1.501 (3)
C8—C9	1.344 (3)	C21—H21A	0.9800
C9—C10	1.437 (3)	C21—H21B	0.9800
C9—H9	0.9500	C21—H21C	0.9800
C10—C11	1.340 (3)	C22—H22A	0.9800
C10—C12	1.478 (3)	C22—H22B	0.9800
C11—H11	0.9500	C22—H22C	0.9800
C12—H12A	0.9900		
			
C11—O1—C8	106.11 (18)	C13—C14—C16	117.97 (17)
C13—O2—C12	117.90 (18)	C15—C14—C16	58.78 (13)
C2—C1—C6	120.7 (3)	C13—C14—H14	115.1
C2—C1—H1	119.7	C15—C14—H14	115.1
C6—C1—H1	119.7	C16—C14—H14	115.1
C3—C2—C1	120.2 (3)	C16—C15—C18	118.33 (18)
C3—C2—H2	119.9	C16—C15—C17	119.24 (19)
C1—C2—H2	119.9	C18—C15—C17	113.2 (2)
C2—C3—C4	120.2 (3)	C16—C15—C14	61.09 (14)
C2—C3—H3	119.9	C18—C15—C14	119.90 (19)
C4—C3—H3	119.9	C17—C15—C14	115.70 (18)
C3—C4—C5	119.7 (3)	C19—C16—C15	122.87 (18)
C3—C4—H4	120.2	C19—C16—C14	118.61 (17)
C5—C4—H4	120.2	C15—C16—C14	60.13 (14)
C4—C5—C6	120.5 (3)	C19—C16—H16	114.8
C4—C5—H5	119.7	C15—C16—H16	114.8
C6—C5—H5	119.7	C14—C16—H16	114.8
C5—C6—C1	118.6 (2)	C15—C17—H17A	109.5
C5—C6—C7	120.7 (2)	C15—C17—H17B	109.5
C1—C6—C7	120.7 (2)	H17A—C17—H17B	109.5
C8—C7—C6	114.2 (2)	C15—C17—H17C	109.5
C8—C7—H7A	108.7	H17A—C17—H17C	109.5
C6—C7—H7A	108.7	H17B—C17—H17C	109.5
C8—C7—H7B	108.7	C15—C18—H18A	109.5
C6—C7—H7B	108.7	C15—C18—H18B	109.5
H7A—C7—H7B	107.6	H18A—C18—H18B	109.5
C9—C8—O1	110.07 (19)	C15—C18—H18C	109.5
C9—C8—C7	133.7 (2)	H18A—C18—H18C	109.5
O1—C8—C7	116.2 (2)	H18B—C18—H18C	109.5
C8—C9—C10	106.8 (2)	C20—C19—C16	127.0 (2)
C8—C9—H9	126.6	C20—C19—H19	116.5
C10—C9—H9	126.6	C16—C19—H19	116.5
C11—C10—C9	105.50 (19)	C19—C20—C21	124.8 (2)
C11—C10—C12	127.2 (2)	C19—C20—C22	120.7 (2)
C9—C10—C12	127.3 (2)	C21—C20—C22	114.5 (2)
C10—C11—O1	111.5 (2)	C20—C21—H21A	109.5
C10—C11—H11	124.2	C20—C21—H21B	109.5
O1—C11—H11	124.2	H21A—C21—H21B	109.5
O2—C12—C10	109.31 (18)	C20—C21—H21C	109.5
O2—C12—H12A	109.8	H21A—C21—H21C	109.5
C10—C12—H12A	109.8	H21B—C21—H21C	109.5
O2—C12—H12B	109.8	C20—C22—H22A	109.5
C10—C12—H12B	109.8	C20—C22—H22B	109.5
H12A—C12—H12B	108.3	H22A—C22—H22B	109.5
O3—C13—O2	123.6 (2)	C20—C22—H22C	109.5
O3—C13—C14	126.8 (2)	H22A—C22—H22C	109.5
O2—C13—C14	109.55 (17)	H22B—C22—H22C	109.5
C13—C14—C15	122.92 (18)		
			
C6—C1—C2—C3	0.2 (4)	C12—O2—C13—O3	0.7 (3)
C1—C2—C3—C4	0.2 (4)	C12—O2—C13—C14	179.52 (18)
C2—C3—C4—C5	−0.8 (4)	O3—C13—C14—C15	−37.8 (3)
C3—C4—C5—C6	1.1 (4)	O2—C13—C14—C15	143.42 (19)
C4—C5—C6—C1	−0.8 (4)	O3—C13—C14—C16	31.4 (3)
C4—C5—C6—C7	−180.0 (2)	O2—C13—C14—C16	−147.39 (18)
C2—C1—C6—C5	0.2 (3)	C13—C14—C15—C16	105.1 (2)
C2—C1—C6—C7	179.3 (2)	C13—C14—C15—C18	−2.8 (3)
C5—C6—C7—C8	−43.0 (3)	C16—C14—C15—C18	−107.9 (2)
C1—C6—C7—C8	137.8 (2)	C13—C14—C15—C17	−144.2 (2)
C11—O1—C8—C9	1.3 (2)	C16—C14—C15—C17	110.7 (2)
C11—O1—C8—C7	179.2 (2)	C18—C15—C16—C19	−142.9 (2)
C6—C7—C8—C9	113.6 (3)	C17—C15—C16—C19	1.7 (3)
C6—C7—C8—O1	−63.7 (3)	C14—C15—C16—C19	106.6 (2)
O1—C8—C9—C10	−1.1 (2)	C18—C15—C16—C14	110.4 (2)
C7—C8—C9—C10	−178.5 (2)	C17—C15—C16—C14	−105.0 (2)
C8—C9—C10—C11	0.5 (2)	C13—C14—C16—C19	133.0 (2)
C8—C9—C10—C12	−179.5 (2)	C15—C14—C16—C19	−113.6 (2)
C9—C10—C11—O1	0.3 (2)	C13—C14—C16—C15	−113.4 (2)
C12—C10—C11—O1	−179.7 (2)	C15—C16—C19—C20	156.9 (2)
C8—O1—C11—C10	−1.0 (3)	C14—C16—C19—C20	−131.9 (2)
C13—O2—C12—C10	121.1 (2)	C16—C19—C20—C21	−0.2 (4)
C11—C10—C12—O2	−123.2 (2)	C16—C19—C20—C22	178.4 (2)
C9—C10—C12—O2	56.8 (3)		

### Hydrogen-bond geometry (Å, º)

Cg is the centroid of the C1–C6 phenyl ring.

**Table:**

*D*—H···*A*	*D*—H	H···*A*	*D*···*A*	*D*—H···*A*
C7—H7*A*···O3^i^	0.99	2.71	3.516 (3)	139
C11—H11···*Cg*^ii^	0.95	2.63	3.559 (3)	167

Symmetry codes: (i) *x*+1, *y*, *z*; (ii) *x*−1, *y*, *z*.

## References

[bb1] Brandenburg, K. (1998). *DIAMOND* Crystal Impact GbR, Bonn, Germany..

[bb2] Bruker (2006). *APEX2* and *SAINT* Bruker AXS Inc., Madison, Wisconsin, USA.

[bb3] Hill, A. S., McAdam, D. P., Edward, S. L. & Skerritt, J. H. (1993). *J. Agric. Food Chem.* **41**, 2011–2018.

[bb4] Sheldrick, G. M. (1996). *SADABS* University of Göttingen, Germany.

[bb5] Sheldrick, G. M. (2008). *Acta Cryst.* A**64**, 112–122. 10.1107/S0108767307043930 18156677

[bb6] Yang, H., Kim, T. H., Park, K.-M. & Kim, J. (2011). *Acta Cryst.* E**67**, o1275. 10.1107/S1600536811014760 PMC3089073 21754558

